# Functional localization and categorization of intentional decisions in humans: A meta-analysis of brain imaging studies

**DOI:** 10.1016/j.neuroimage.2021.118468

**Published:** 2021-11-15

**Authors:** Ruoguang Si, James B Rowe, Jiaxiang Zhang

**Affiliations:** a Cardiff University Brain Research Imaging Centre, School of Psychology, Cardiff University, Cardiff CF24 4HQ, United Kingdom; b Department of Clinical Neurosciences, University of Cambridge, Cambridge CB2 0QQ, United Kingdom; c Medical Research Council Cognition and Brain Sciences Unit, University of Cambridge CB2 7EF, United Kingdom

**Keywords:** Intentional decision, Free choice, Meta-analysis, ALE, fMRI, PET

## Abstract

Brain-imaging research on intentional decision-making often employs a “free-choice” paradigm, in which participants choose among options with identical values or outcomes. Although the medial prefrontal cortex has commonly been associated with choices, there is no consensus on the wider network that underlies diverse intentional decisions and behaviours. Our systematic literature search identified 35 fMRI/PET experiments using various free-choice paradigms, with appropriate control conditions using external instructions. An Activation Likelihood Estimate (ALE) meta-analysis showed that, compared with external instructions, intentional decisions consistently activate the medial and dorsolateral prefrontal cortex, the left insula and the inferior parietal lobule. We then categorized the studies into four different types according to their experimental designs: reactive motor intention, perceptual intention, inhibitory intention, and cognitive intention. We conducted conjunction and contrast meta-analyses to identify consistent and selective spatial convergence of brain activation within each specific category of intentional decision. Finally, we used meta-analytic decoding to probe cognitive processes underlying free choices. Our findings suggest that the neurocognitive process underlying intentional decision incorporates anatomically separated components subserving distinct cognitive and computational roles.

## Highlights

• We performed meta-analyses on fMRI/PET studies of human intentional decision.
• Intentional choices activate a brain network maximal in the medial frontal cortex.
• Four types of intentional decision paradigms are identified in the literature.
• Intentional decisions rely on regions with distinct cognitive and computational roles.

## 1 Introduction

To fulfil our goals or desires, we constantly interact with the external environment through our voluntary behaviour. In contrast to reflexes that are beyond volition (e.g., a knee-jerk reflex), voluntary behaviours are characterised by choice (Passingham, 1995). Volition characterises the intentional choice or decision between multiple options, where the choice is not sufficiently explained by differences in expected or explicit rewards. The concept of intentional decision refers to this fundamental ability of human cognition: acting voluntarily based on internal or endogenous intentions (Marken, 1982).

The role of intention in decision-making occupies a broad spectrum. At one extreme lies externally guided perceptual decision such as stopping at a red traffic light, for which the involvement of internal intention is low because learned rules can dictate a correct choice (even if one can voluntarily break such rules). At the other extreme lies improvisational behaviour in music, painting or dance, which can be strongly determined by moment-to-moment intention. In between lies the common scenario of intentional decision-making, where the external environment constrains only which options are available while internal intentions dictate which of those options to choose. The ability to choose actions, cognitive strategies and behaviours in this way plays a key role throughout the life span and is essential to our understanding of human cognition. In child development from birth to 12 months, actions such as grasping and its coordination with vision gradually emerge from simple reflexes (Piaget, 1976; Beilin and Fireman, 1999; Lewis, 2010). In patients with neurodegenerative disorders, the inability to engage appropriate intentional behaviour can manifest as apathy (Starkstein et al., 2001), impulsivity (Dalley et al., 2011) and perseveration (Hughes et al., 2013). In addition, intentional behaviour is a foundation of social interactions via cooperation and collaboration (Bratman, 2017).

Intentional actions have been characterised by three components in the *what*-*when*-*whether* (WWW) model: (1) *what* action to perform, (2) *when* to perform it, and (3) *whether* to perform the chosen act (Brass and Haggard, 2008). The WWW model is based on evidence from two interlinked lines of research. First, the *when* component has been investigated by examining neural signatures immediately prior to intentional actions. Libet's intentional action paradigm is a classic example of this type (Libet et al., 1983; Libet, 1985), which has been used to localize electrophysiological and BOLD activity in the medial-frontal cortex preceding the conscious awareness of subsequent voluntary actions (Lau et al., 2004a; Fried et al., 2011) (but see Trevena and Miller, 2002; Nachev and Hacker, 2014 for critical evaluations). Second, research on the *what* and *whether* components, the focus of the current study, commonly use variants of the “free-choice” paradigm to determine the neurocognitive mechanisms of voluntary decision processes. In the literature, several terms have been used to refer to the free-choice paradigm, such as “voluntary selection” (Forstmann et al., 2006), “willed action” (Lau et al., 2004b), “internal selection” (Van Oostende et al., 1997), “self-initiated” (Cunnington et al., 2002), and “chosen actions” (Zhang et al., 2012). The current study uses these terms interchangeably.

In a typical free-choice paradigm, participants make a voluntary choice from multiple alternatives on each trial. The available alternatives can either be similar to each other (e.g., responding with different fingers, Zhang et al., 2012) or distinct (e.g., to choose voluntarily between stopping and acting in the adapted Go/NoGo task, Karch et al., 2009). Importantly, participants are made aware that all available options are homogeneous in terms of their objective outcomes, and the tasks do not introduce or manipulate rewards of costs according to the choices made. In other words, the task is not to identify a correct response. Rather participants can choose any of the available options. The alternate options are equally appropriate, and one's decision must come from intention. The intention could be influenced by endogenous factors, including subtly differential effort, preferences (Zajkowski et al., 2020), habits (Graybiel, 2008), incorrectly inferred arbitrary rules for the task, and recent actions (Zhang and Rowe, 2015; Phillips et al., 2018).

In recent years, there has been a substantial number of brain imaging studies adopting free-choice paradigms, enabling a well-powered meta-analysis. The current study focused on the hemodynamic and metabolic contrasts of intentional choice vs. specified response, which is the most widely reported task-related effect across free-choice studies. Here, specified responses serve as a control condition, in which participants need to make specific responses determined by the experimenter, rather than choose voluntarily from the same set of options in the free-choice condition. Therefore, the contrast between the two conditions offers an imaging marker of brain activation associated with intentional behaviour, controlling for the common effects of stimulus encoding and response initiation.

The objectives of this study were three-fold. First, to identify brain regions consistently activated by intentional decision, we performed a systematic search of BOLD-fMRI or PET studies of intentional decision and conducted an activation likelihood estimation (ALE) meta-analysis. Increased BOLD-fMRI and PET responses during intentional choices are commonly reported in a frontoparietal network centred on the medial frontal cortex (Brass and Haggard, 2008). However, some studies also observed activations external to this network during intentional behaviour, in particular in the insula (Brass and Haggard, 2010; Thimm et al., 2012; Dall'Acqua et al., 2018) and the inferior frontal gyrus (Wisniewski et al., 2016). Because results from a coordinate-based ALE meta-analysis are pooled from a large number of participants in multiple studies, they usually have higher statistical power than a single experimental study (Walker et al., 2008). Using ALE analyses, we aim to test whether there are significant clusters of foci associated with intentional decision across a wide range of free-choice paradigms, and whether these clusters extend beyond the commonly reported medial prefrontal cortex.

**Fig. 1:**
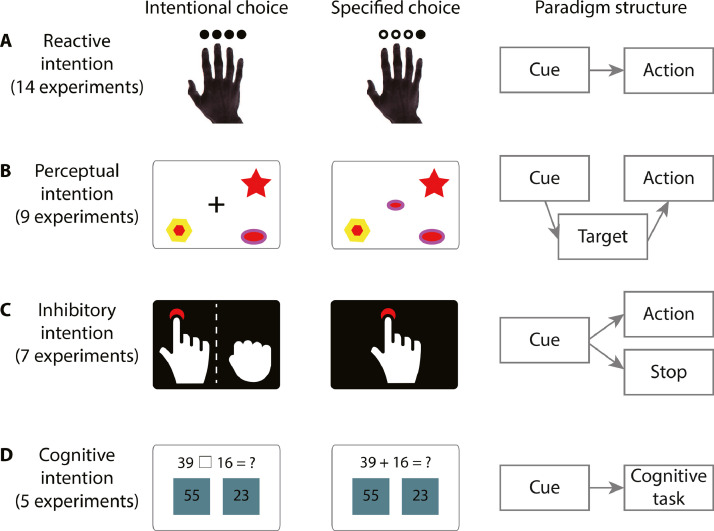
Schematics and examples of four categories of free-choice studies. (**A**) In the reactive intention (RI) paradigm, task cues directly indicate available actions. For example, in Rowe et al. (2008), the cue for intentional choice was four black dots, indicating that participants can respond with any one of their four fingers. The cue for a specified response was a black dot over a specific finger, indicating that participants should only respond with that finger. (**B**) In the perceptual intention (PI) paradigm, task cues contain perceptually similar options that associated with different options. For example, in Lau et al. (2004), the cue for intentional choice was the cross at the middle of the screen, indicating that participants can choose any of the patterns on the screen. The cue for a specified response was a specific pattern at the centre of the screen, indicating that participants should choose the pattern that matches the cue. (**C)** In the inhibitory intention (II) paradigm, one of the options is to abandon or abort an intended action. Hence, participants make voluntary choices between Go and Stop (e.g., Dall'Acqua et al., 2018). In the corresponding specified response condition, participants are instructed to either execute or inhibit their actions. (**D**) In the cognitive intention (CI) paradigm, participants choose between different operations that require higher-level cognitive processing. Behavioural responses are dependent on the execution of the chosen cognitive operation. For example, in Wisniewski et al. (2016), the cues were different for the intentional and specified condition. In the intentional condition, participants chose to add or subtract two numbers and then select the correct answer based on their choice. In the specified condition, participants followed the instruction to perform a specific arithmetic operation.

Second, we conducted further contrast and conjunction meta-analyses, assessing the differential and common convergence of brain activations reported by studies investigating different types of intentional behaviour. As highlighted above, the nature of options in a free-choice paradigm can vary significantly between studies and hence involve different cognitive processes. We reviewed all studies to date that met our predefined inclusion criteria (see *Study selection and inclusion criteria*). Based on the experimental design and implementational details of individual studies, we proposed four categories of the free-choice paradigm (Fig. 1, see *Paradigm-specific meta-analysis* for the definition of each paradigm): reactional intention (RI), perceptual intention (PI), inhibitory intention (II) and cognitive intention (CI). We hypothesize significant clusters of foci associated with each category of free-choice paradigms. Using ALE contrast analysis, we further tested the hypothesis that some brain regions are more likely to be activated in one free-choice paradigm over another.

Third, to undertake an exploratory data-driven analysis, testing whether consistent BOLD-fMRI/PET patterns of intentional behaviour correspond to specific cognitive processes. We quantified the similarity between the meta-analytical whole-brain activation pattern estimated from free-choice studies and brain activation patterns from 100 specific cognitive topics, extracted from a database of over 11,000 brain imaging studies (Yarkoni et al., 2011; Rubin et al., 2017). This “decoding” approach raises hypotheses about the putative cognitive processes underpinning intentional behaviour, where different cognitive processes are associated with specific networks of the human brain. Finally, we reviewed results from these meta-analyses in the context of current cognitive models of intentional choice.

## 2 Materials and methods

### 2.1 Study selection and inclusion criteria

We defined intentional choices as experimental paradigms involving self-initiated, voluntary selections of an action from two or more alternatives (Zhang et al., 2012). The experimental procedure would need to instruct participants that there are no correct or incorrect choices, and they are free to choose any option among available alternatives. This type of intentional choices differs from conventional goal-directed or externally cued behaviour, in which a correct or instructed response could be defined or identified. We focused on existing studies investigating the *“what”* (which action to choose) or “*whether*” (whether or not to execute an action) component of intentional behaviour (Brass and Haggard, 2008). Studies focusing on the “*when*” component (i.e., when to execute, as in Libet et al., 1983) is not considered here, but is briefly reviewed in Discussion.

We conducted a systematic literature search in accordance with the PRISMA guidelines (Moher et al., 2015) to identify brain imaging studies of intentional choice. The literature search was performed on both PubMed and PubMed Central (PMC) databases, because the two databases may contain different publications. The PubMed database was searched with specified keywords as following: ("volitional decision" OR "volitional choice" OR "voluntary decision" OR "intended decision" OR "intentional decision" OR "voluntary choice" OR "intended choice" OR "intentional choice" OR "free decision" OR "free choice" OR "volitional action" OR "voluntary action" OR "intended action" OR "intentional action" OR "free action" OR "volitional selection" OR "voluntary selection" OR "intended selection" OR "intentional selection" OR "free selection") AND ("fMRI" OR "functional Magnetic Resonance Imaging" OR "BOLD" OR "Blood Oxygen Level-Dependent" OR "Positron Emission Tomography"). For the PMC database, the same keywords were employed in the interrogation and a filter on the search field was set to “Body - Key Terms” to constrain search in a more concrete range. The search results from PubMed and PMC databases were combined with duplicated records removed, resulting in 332 publications as of October 2020.

We then inspected every publication from the literature search. The further inclusion criteria for our meta-analysis were applied as follows:

1 Studies reported first hand data that comes from experiments rather than reviews or meta-analysis. 291 of the 332 publications met this criterion.
2 Studies included results from healthy adult participants. 239 of the remaining 291 publications met this criterion.
3 Studies employed an intentional choice paradigm(s) and reported a fMRI/PET contrast of intentional choice vs. specified response conditions. Here, in the specified response condition, participants responded with the same set of possible actions as in the intentional choice condition, but the identity of which action to respond (or whether to respond) was determined by the experimenter. 37 of the remaining 239 publications met this criterion.
4 Studies reported whole-brain analysis with MNI or Talairach coordinates of the cluster peaks. 34 of the remaining 37 publications met this criterion.
5 If more than one appropriate contrast with the same group of subjects were reported in a single study, only one contrast was included in the meta-analysis.

### 2.2 Activation likelihood estimation (ALE) meta-analysis of intentional decision

After the screening, 34 fMRI/PET studies met the selection criteria, which included 35 independent experiments for meta-analysis. 25 studies recruited only right-handed participants, 1 study recruited thirteen right-handed and one left-handed participants, and the other 8 studies did not specify participants’ handedness. These studies contained a total of 633 participants and reported 329 peak foci of increased fMRI/PET responses to the intentional choice vs. specified response contrast. Less than 3% foci (9 out of 329) were out of the brain mask in the GingerALE toolbox (Turkeltaub et al., 2002; Eickhoff et al., 2012), which was within the normal range due to spatial smoothing and potential registration errors (Eickhoff et al., 2012). Therefore, all foci were included in the study to maximize the usage of the original dataset. For activation foci reported in the Talairach space, we converted them to MNI coordinates using the Lancaster algorithm (Lancaster et al., 2007).

The coordinates-based activation likelihood estimation (ALE) meta-analysis was conducted over all the 35 experiments using the Ginger-ALE toolbox (www.brainmap.org, version 3.0.2) (Turkeltaub et al., 2002; Eickhoff et al., 2012). This analysis aimed to determine, across independent experiments, significant spatial convergence of fMRI/PET activation probabilities for the intentional choice vs. specified response contrast, under the null hypothesis that the activation foci are distributed randomly throughout the brain. First, for each experiment, the activation probabilities of all foci reported were modelled as 3D Gaussian probability distributions with their full-width half-maximum (FWHM) estimated from the between-subject variance of the experiment (Eickhoff et al., 2009). Second, an ALE activation map was then calculated by combining all experimental-level activation maps, yielding a voxel-wise ALE score to quantify the convergence of results across experiments at each voxel location. Third, an analytical approach was used to determine the null distribution of voxel-wise ALE scores. A non-parametric *p*-value map of ALE scores was then generated under the null distribution (Eickhoff et al., 2012). Finally, the *p*-value map was thresholded at *p <* 0.001 and corrected for multiple comparisons across voxels using a cluster-level family-wise error (FWE) correction from 5,000 permutations (*p* < 0.01, cluster-corrected).

### 2.3 Paradigm-specific meta-analysis

We categorized the 35 experiments into four intentional choice paradigms based on their experimental designs and procedures (Fig. 1). The first category is referred to as reactional intention (RI), in which participants voluntarily choose cues that associate to specific motor actions. We considered this category as the simplest form of intentional choice because a cue in the RI paradigm is directly linked to a target action. The second category is referred to as perceptual intention (PI), in which participants voluntarily choose between perceptually distinct targets (e.g., icons or pictures). Compared to the RI paradigm, the PI paradigm involves an additional matching process: an option is associated with a perceptual target, and the target is then associated with a specific motor action. The third category is referred to as inhibitory intention (II), in which at least one option is not to act (i.e., withholding responses). A cue in the II paradigm is associated directly with a specific action or the inhibition of action. The final category is referred to as cognitive intention (CI). The free choice condition in the CI paradigm requires the participants to choose between options that involve higher-order cognitive processes such as doing arithmetic or generating words.

For studies employed each of the four paradigm categories, we performed the same ALE meta-analysis to identify the spatial convergence of fMRI/PET activation for the intentional choice vs. specified response contrast. The same procedure to correct for multiple comparisons was applied as in the meta-analysis across all studies (see Section 2.2).

Based on the thresholded ALE maps from individual paradigms, we then conducted further conjunction and contrast meta-analyses between the RI and PI paradigms as well as the RI and II paradigms, using the “contrast study” function implemented in GingerALE. This allowed us to localize voxels commonly (i.e., conjunction) or differentially (i.e., contrast) activated across intentional choice paradigms. The conjunction images were created using the voxel-wise minimum value of the input ALE images. To correct for study sizes (Eickhoff et al., 2011), the contrast analyses were conducted through permutation tests. First, the ALE differences image was created by directly subtracting one input image from the other. Second, the simulated data was created by pooling the foci datasets and randomly dividing them into two new groups with the same size as the original groups. Third, a new ALE difference image was created by directly subtracting of the two new datasets and then compared to the true data. Fourth, with multiple permutations, a voxel-wise *p-*value image was created to illustrate where the true data sit on the distribution of the ALE differences in each voxel. A lenient threshold (cluster threshold 200 mm^3^, uncorrected voxel-level threshold *p* < 0.01, permutation tests with 5,000 iterations) was applied to the contrast analyses between paradigm categories to avoid type II errors (Lieberman and Cunningham, 2009). No conjunction or contrast meta-analysis was conducted on experiments using the CI paradigm due to the limited number of studies available in that category.

### 2.4 Meta-analytic decoding of intentional decision

ALE activation maps indicate brain regions of consistent fMRI/PET activations between studies. We then used NeuroSynth (Yarkoni et al., 2011) to perform a “reverse-inference” type of meta-analysis. That is, we meta-analytically decoded which cognitive functions or processes are likely to give rise to the consistent brain activations observed in ALE activation maps. As highlighted previously, one should interpret results from reverse inference with caution (Poldrack, 2011). Most functional brain imaging results are correlational. The involvement of a brain region in a certain cognitive function does not directly support the notion that the region is exclusively associated with the cognitive process. Nevertheless, meta-analytic decoding against large, unbiased imaging databases did provide useful information about the engagement of cognitive processes (Poldrack, 2006). In the current study, we consider our meta-analytic decoding analysis to be contributory rather than confirmatory, which offers insights for future studies of intentional decision.

We considered a set of 100 cognitive topics that were previously generated from over 11,000 brain imaging studies. The 100 topics were extracted by fitting a generative statistical model of sematic topics (Blei et al., 2003) to the abstracts of over 11,000 brain imaging articles in the NeuroSynth database (for details see Poldrack et al., 2012). We ignored the topics related to general methods (e.g., fMRI) and focus only on the topics related to cognitive processes. For each cognitive topic, a whole-brain association-test map (also referred to as the reverse inference map) was generated from all the articles in the database. The value at each voxel of the association-test map quantifies the extent to which studies loaded highly on the current topic reported more consistent activation at this location than all the other studies (Yarkoni et al., 2011).

We estimated the similarity between each unthresholded ALE activation *p-*value map with respect to the association-test maps of the 100 cognitive topics by calculating their Pearson correlations across voxels. The resulting correlation coefficients were rank ordered to identify the cognitive topics that are most likely to be present during intentional decision and its specific paradigms.

### 2.5 Data availability statement

All data used in this meta-analysis study were obtained from original publications. We have made the aggregated data open access (https://osf.io/bhwj5), which include the imaging data entered meta-analyses and unthresholded statistical maps from meta-analyses.

### 2.6 Ethics statement

This study did not include data from new participants. In all meta-analyses, we only considered studies that had obtained informed consent from human participants.

## 3 Results

### 3.1 Meta-analysis of intentional decision

Thirty-four brain imaging studies were identified from our symmetric literature search, which included 35 independent experiments of intentional decision. The number of participants, experimental paradigms and other details were summarized in Table 1.

**Table 1:** List of intentional decision studies that meet the inclusion criteria.

No.	Study	Number of subjects	Imaging modality	Experiment Paradigm	Contrast used in meta-analyses
1	(Beudel and De Jong, 2009)	16	fMRI	RI: Watch number cues to press button using the 2nd to the 5th finger of right hand	Table 1, B. II. finger selection, free versus fixed condition
2	(Deiber et al., 1991)	8	PET	PI: Listen auditory cue to push the joystick to different direction	Table 1. random vs. fixed condition
3	(Deiber et al., 1996)	13	PET	PI: Watch light cue to abduct or elevate the index or little finger with right hand	Table 3, free vs. full condition
4	(François-Brosseau et al., 2009)	14	fMRI	RI: Watch square colour change to press button with 2nd to 5th finger of one hand▲	Table 3. self-initiated vs. externally-triggered movements, right hand
5	(Frith et al., 1991)	6	PET	RI: Feel the touch cue to lift the 1st or 2nd finger of the right hand	Table 2, study 2, task 3 (free) - task 1 (specified), increased
6	(Gerardin et al., 2004)	9	fMRI	RI: Press button with left or right thumb▲	Table 1, right hand, select vs. prepare
7	(Hoffstaedter et al., 2013)	35	fMRI	RI: Watch arrow cue to press button with right or left index finger	Table S1, Timed vs. No Choice
8	(Hyder et al., 1997)	9	fMRI	RI: Feel the touch cue to move the first or second finger of the right hand (the paradigm is similar to the PET study by Frith et al., 1991)	Table 1. random vs. repeat
9	(Krieghoff et al., 2009)	16	fMRI	RI: Watch letter cue to press left or right button with the index finger of one hand	Table 1, cue-related activation, internal > external
10	(Mueller et al., 2007)	16	fMRI	RI: Watch visual cue tto press right or left button with the index finger of right hand	Table 1, internally vs. externally selected actions
11	(Rae et al., 2014)	17	fMRI	RI: Watch circle color change to press button with 2nd to 5th finger of right hand	Table S2, action selection (go select > go specified)
12	(Rowe et al., 2010)	20	fMRI	RI: Watch circle colour change to press button with 2nd to 5th finger of right hand	Table 1, chosen vs. specified responses
13	(Schouppe et al., 2014)	22	fMRI	RI: Watch arrow cue to choose the right or left direction (adapted flanker task)	Table 2, voluntary vs. imposed choice
14	(Van Eimeren et al., 2006)	12	fMRI	RI: Watch the circle brightness change to press button with 2nd or 3rd finger of both hands	Table 1, selection vs. non-selection
15	(Bode et al., 2013)	15	fMRI	PI: Choose picture by button pressing	Table 2, free decision vs. high visibility condition
16	(Filevich et al., 2013)	23	fMRI	PI: Choose number with mouse cursor	Table 1, free vs. instructed
17	(Forstmann et al., 2006)	22	fMRI	PI: Choose target by button pressing	Table 1, main contrast of choice
18	(Lau et al., 2004b)	12	fMRI	PI: Choose target pattern with cursor	Table 1, free vs. specified
19	(Orr and Banich, 2014)	28	fMRI	PI: Choose task by button pressing	Table 1, voluntary vs. explicit
20	(Rens et al., 2018)	24	fMRI	PI: Choose target door by button pressing	In text, choice stay vs no-choice Stay
21	(Rowe et al., 2005)	12	fMRI	PI: Choose target by button pressing	Table 2, Combined colour and action tasks
22	(Rowe et al., 2008)	20	fMRI	PI: Choose action by button pressing	Table 3, All free-specified
23	(Thimm et al., 2012)	28	fMRI	PI: Press target button by analysing colour or position cues	Table 1, free vs. specified choice
24	(Dall'Acqua et al., 2018)	24	fMRI	II: Adapted go/no-go paradigm*	Table 2, free-choice vs. cued
25	(Karch et al., 2010b)	8	fMRI	II: Adapted go/no-go paradigm*	Table 4, [(selection- + selection+)– (go + no-go)] in healthy controls
26	(Karch et al., 2010a)	15	fMRI	II: Adapted go/no-go paradigm*	Table 1, Voluntary selection > control
27	(Karch et al., 2009)	14	fMRI	II: Adapted go/no-go paradigm*	Table IV, voluntariness
28	(Lynn et al., 2016)	21	fMRI	II: Pain stop or endurance by button pressing or not	Table 1, Main effect choice: choice > directed
29	(Omata et al., 2019)	26	fMRI	II: Whether to stop the continuous finger-tapping	Table 1, voluntary stop - forced stop
30	(Schel et al., 2014)	24	fMRI	II: Adapted go/no-go paradigm*	Table 1, conjunction intentional action and inhibition
31	(Frith et al., 1991)	6	PET	CI: Generate word or repeat word	Table 2, study 1, task 3 (free) - task 1 (specified), increased
32	(Jarvstad and Gilchrist, 2019)	23	fMRI	CI: saccadic selection	Table 2, choice (choice vs. low)
33	(Ort et al., 2019)	22	fMRI	CI: Redirect attention to target(s) without actual movement	Table S3, Proactive Events > Reactive Events (collapsed across Trial Transition)
34	(Taylor et al., 2008)	18	fMRI	CI: Redirect attention to target(s) without actual movement	Table 1, choice vs. instructed
35	(Wisniewski et al., 2016)	35	fMRI	CI: mathematical calculation (subtract or addition)	Table 1, free vs. cued
Total	/	633	/	/	/

⁎ The adapted go/no-go task includes intentional trials in addition to conventional go/no-go trials. In each intentional trial, participants were free to choose whether to respond.

▲ The study reported the contrast of intentional decision and specified response separately for left and right hands. Only the results from the dominant hand (right hand) were included in the meta-analysis.

There is no correct answer among available options in free choice paradigms. Nevertheless, behavioural performance can be evaluated in other ways, for example using response time (normally referred to the latency between task cues and responses) and the proportion of valid response. The latter applies to experiments (e.g., Zhang et al., 2012) in which participants make a free choice from *M* available options when there is a total of *N* options (where *M*<*N*). If an unavailable option was chosen, the response is deemed to be invalid. Here, the proportion of valid response is akin to the conventional decision accuracy. Supplementary Table 3 summarized the behavioural responses from studies included in the meta-analysis. The general pattern of the high proportion of valid response and fast RT suggest that participants did actively perform tasks in those studies.

**Fig. 2:**
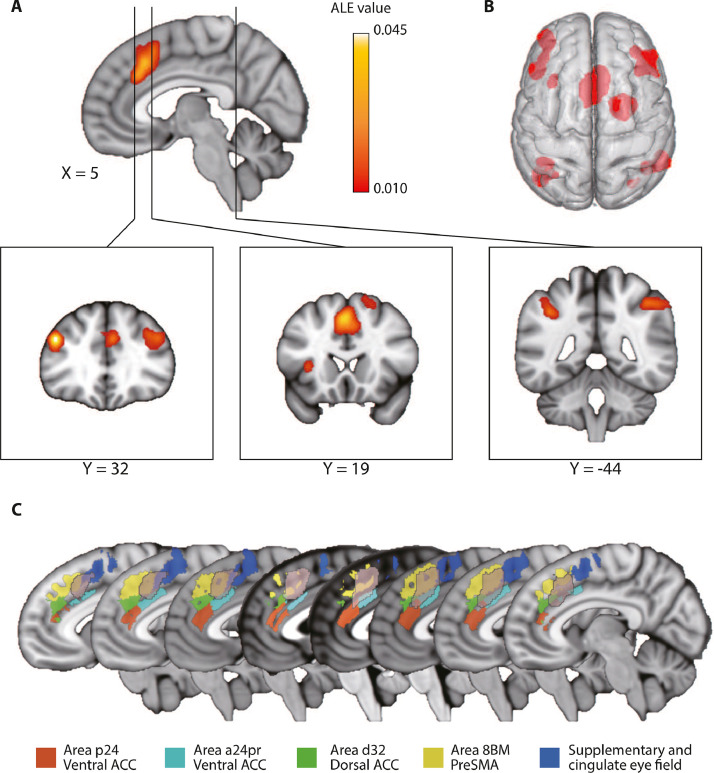
ALE meta-analyses of all free-choice studies showing significant clusters related to intentional decision (*p*<0.01, cluster-level FWE corrected from 5,000 permutations). (**A**) ALE value map. (**B**) 3D render of all the clusters. Table 2 lists the peak coordinates of each cluster. (**C**) Spatial extent of the ACC/pre-SMA cluster overlayed with the medial-prefrontal ROIs from the HCP-MMP atlas (Glasser et al., 2016). The x-coordinates of the sagittal slices are from -7 mm (left) to 7 mm (right), with a step size of 2 mm in adjacent slices.

Across all the 35 experiments, a Ginger-ALE meta-analysis on the contrast between free choice and specified response yielded greater BOLD-fMRI/PET activations related to intentional behaviour in a frontoparietal network (Fig. 2). The analysis identified 19 peaks in 7 clusters, including bilateral pre-supplementary motor area (pre-SMA), bilateral anterior cingulate cortex (ACC), bilateral dorsal lateral prefrontal cortex (dlPFC), bilateral inferior parietal lobule (IPL), right premotor area and left anterior insula cortex (AIC) (Table 2, *p* < 0.01, cluster-level corrected). At a more lenient threshold (*p* < 0.01 uncorrected), there was a cluster in the right AIC (peak coordinate: *X* = 34, *Y* = 24, *Z* = 8).

**Table 2:** Meta-analysis results of intentional decision (“free choice” > “specified response”) across all studies. Peak coordinates of clusters were reported in the MNI space (mm).

Cluster	Label	X	Y	Z	ALE score (max)	Cluster size (mm^3^)
1	Left pre-SMA	-2	20	46	0.041007	9424
	Right ACC	6	24	40	0.036621	
	Right ACC	8	28	34	0.036324	
2	Left dlPFC	-42	32	30	0.047762	6224
		-38	50	10	0.030668	
		-40	38	22	0.020322	
3	Right dlPFC	46	40	22	0.029948	6192
		44	34	32	0.027517	
		34	42	18	0.027402	
4	Right IPL	54	-38	46	0.030843	4504
		44	-46	48	0.026652	
		32	-62	44	0.020985	
		38	-54	50	0.014810	
5	Right Premotor	28	10	52	0.031123	4128
		16	14	64	0.026349	
6	Left IPL	-42	-52	50	0.030874	3824
		-40	-44	40	0.027230	
		-30	-52	44	0.017769	
7	Left Insula	-34	14	2	0.028333	1208

**Table 3:** Meta-analysis results of individual paradigms of intentional decision. Peak coordinates of clusters were reported in the MNI space (mm).

Cluster	Label	X	Y	Z	ALE score (max)	Cluster size (mm^3^)
**Reactive Intention**						
1	Right pre-SMA	2	18	50	0.021743	3800
	Right FEF	8	24	42	0.018563	
	Right ACC	4	28	30	0.011148	
2	Left rlPFC	-36	44	14	0.016166	2368
	Left dlPFC	-44	30	24	0.015478	
		-36	38	22	0.012896	
3	Left IPL	-38	-44	38	0.019811	1760
		-50	-40	42	0.011834	
4	Right Premotor	24	4	56	0.013911	1440
		18	20	58	0.013302	
5	Right IPL	54	-36	50	0.017881	1376
6	Right dlPFC	36	28	34	0.014104	1096
		42	32	26	0.010655	
**Perceptual Intention**						
1	Left PreSMA	-2	22	48	0.021206	2320
	Left ACC	-2	26	38	0.015668	
2	Left dlPFC	-44	30	30	0.019158	920
		-46	20	30	0.008827	
3	Left rlPFC	-40	52	2	0.011520	800
		-34	60	-8	0.009771	
		-42	50	-6	0.008851	
4	Right dlPFC	44	36	34	0.013186	784
**Inhibitory Intention**						
1	Left IPL	-44	-48	50	0.019167	1600
		-54	-42	50	0.008922	
2	Right IPL	52	-44	46	0.017142	1536
3	Right Premotor	28	12	56	0.014202	1272
		18	16	64	0.013301	
4	Right dlPFC	44	40	22	0.012603	768
		36	32	26	0.009217	
5	Left rlPFC	-38	50	6	0.011002	752
		-32	56	14	0.010811	
		-30	52	12	0.010744	
6	Right pre-SMA	4	28	50	0.011269	744
		8	24	42	0.009571	
		2	18	42	0.009109	
7	Left dlPFC	-42	32	30	0.015433	656

The medial prefrontal cluster from ALE meta-analysis extends from *Y* = 6 (posterior) to *Y* = 34 (anterior). Voxels in this cluster mainly intersect with one of the five ROIs in the HCP-MMP atlas (Glasser et al., 2016): a24pr (ventral ACC), p24 (ventral ACC), d32 (dorsal ACC), 8BM (pre-SMA) and SCEF (supplementary and cingulate eye fields) (Fig. 2C). The posterior boundary of the medial prefrontal cluster falls in the SCEF (Brodmann area 6).

### 3.2 Meta-analysis of contrasts between intentional decision paradigms

To investigate whether different types of intentional behaviour relate to selective brain responses, we assigned intentional decision studies into four categories (Fig. 1), depending on their characteristics of experimental paradigms: reactive intention (RI), perceptual intention (PI), inhibitory intention (II) and other higher cognitive intention (CI).

Free choices in the RI paradigm were consistently associated with greater activations in 6 clusters with 13 peak foci, including bilateral pre-SMA and ACC, bilateral IPL, bilateral prefrontal area and right premotor area. For the PI paradigm, the analysis revealed 4 clusters with 8 peak foci located in bilateral prefrontal area, left ACC and pre-SMA cluster. For the II paradigm, there were 7 clusters with 14 peak foci located in bilateral IPL, right premotor area, bilateral prefrontal area and right PreSMA. No significant results were observed in the meta-analysis of the CI paradigm, possibly due to the limited number of studies in that category.

**Fig. 3:**
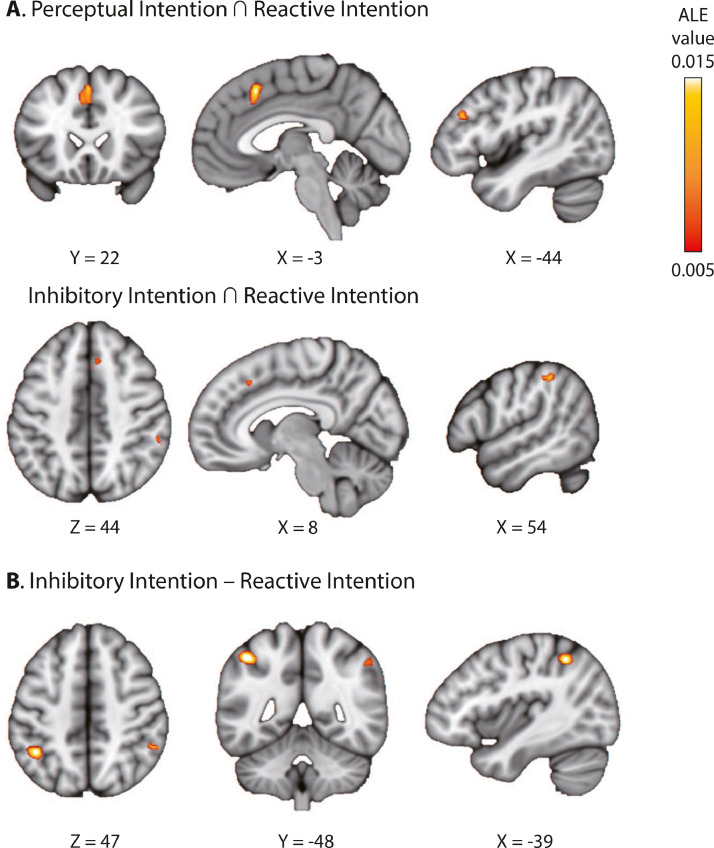
Contrast and conjunction meta-analyses (**A**) ALE conjunction meta-analyses between PI and RI paradigms (top) as well as II and RI paradigms (bottom). (**B**) ALE contrast meta-analyses between II vs. RI paradigms. Table 4 lists the peak coordinate of each cluster.

To quantify the differential and common convergence of brain activation in studies of different types of intentional behaviour, we conducted further contrast and conjunction meta-analyses, comparing both the PI paradigm (involving perceptual processing) and the II paradigm (involving inhibitory processing) with the most elementary paradigm (i.e., the RI paradigm). The contrast meta-analysis showed that the bilateral IPL is more likely to activate in the II than the RI paradigm (Fig. 3B, Table 4). No significant difference was found between PI and RI paradigm. The conjunction meta-analysis showed that bilateral Pre-SMA/ACC complex and the left dlPFC are commonly activated in intentional behaviour across studies of PI and RI paradigms, and activations in the right IPL and right PreSMA are commonly observed in both II and RI paradigms (Fig. 3A, Table 4).

**Table 4:** Contrast and conjunction meta-analyses between different free-choice paradigms. Peak coordinates of clusters were reported in the MNI space (mm).

Cluster	Label	X	Y	Z	ALE score	Cluster size (mm^3^)
**Inhibitory Intention > Reactive Intention**						
1	Left IPL	-42	-50	50	0.018786	1224
2	Right IPL	52	-46	46	0.013505	288
**Perceptual Intention ∩ Reactive Intention**						
1	Left pre-SMA/ACC	-2	18	48	0.015814	1104
2	Left dlPFC	-44	32	28	0.012595	256
**Inhibitory Intention ∩ Reactive Intention**						
1	Right IPL	54	-38	46	0.012834	344
2	Right Pre-SMA	8	24	42	0.009571	216
		2	18	42	0.009109	

### 3.3 Meta-analytic decoding of intentional decision

To probe cognitive processes underlying intentional decision, we assessed the spatial similarity (i.e., Pearson correlation across voxels) between the ALE activation maps from our meta-analysis and 100 association-test maps. Each of the association-test maps represents brain response selective to one of 100 psychological topics, generated from meta-analyses of >11,000 independent studies (Yarkoni et al., 2011). Therefore, a high correlation coefficient to an association-test map would imply the potential involvement of the corresponding cognitive process. The primary interest here is the relative ranking of the topics based on the similarity of their association-test maps to our results, not to perform null hypothesis significance testing on each correlation.

**Fig. 4:**
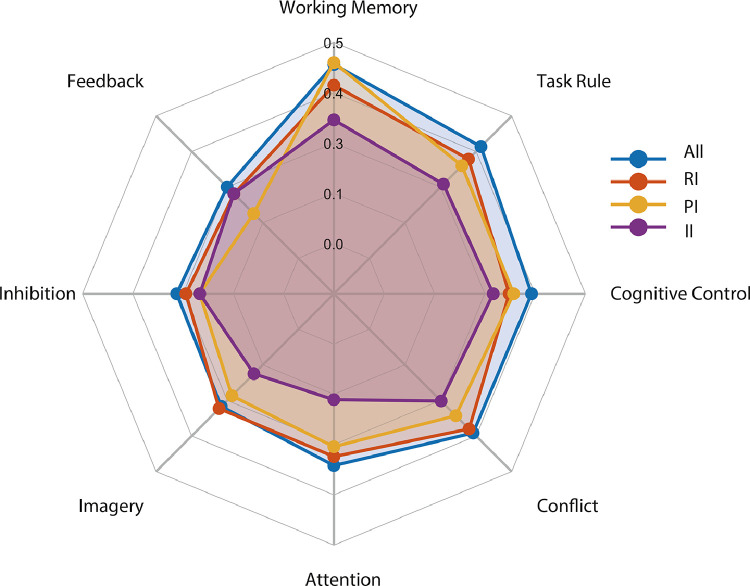
Meta-analytic decoding of intentional decision. Correlation coefficients between different cognitive topics’ association maps and ALE maps of intentional decision were calculated. The correlation values for the top 8 topics were illustrated in a polar plot. Terms used to generate those topic-based association maps were listed in Supplementary Table 1.

This exploratory analysis showed that the frontoparietal network identified in the meta-analysis of intentional decision across all studies (Fig. 4) was strongly associated with several psychological topics. The top three are working memory (*R* = 0.445), task rules (*R* = 0.392) and cognitive control (*R* = 0.366) (Fig. 4, and see Supplementary Tables 1 and 2 for full results).

We also applied the same decoding procedure to paradigm-specific ALE-activation maps. The RI paradigm was associated with the topic of working memory (*R* = 0.393), conflict (*R* = 0.350) and task rules (*R* = 0.348). PI paradigm was associated with the topic of working memory (*R* = 0.450), task rules (*R* = 0.325) and cognitive control (*R* = 0.322). II paradigm was associated with the working memory (*R* = 0.307), cognitive control (*R* = 0.271), task rules (*R* = 0.260) and feedback (*R* = 0.227).

## 4 Discussion

Our meta-analysis considered the convergence of fMRI/PET responses reported in intentional decision studies. Across a large variety of free-choice paradigms, when contrasted against the same behavioural response prescribed exogenously (Fig. 2), intentional decision is associated with a convergence of BOLD/PET activity in the medial prefrontal cortex (pre-SMA and caudal ACC), the lateral frontoparietal cortices (DLPFC and IPL) and the anterior insula cortex (AIC). Here, we specifically focused on the free-choice paradigm, in which participants were given the freedom to choose among multiple options, instead of seeking a correct answer as in perceptual or reward based decision-making tasks. Our study further delineates a broad spectrum of free-choice paradigms in the current literature, with category-specific ALE results.

Previous meta-analysis studies on similar topics have distinct research questions. For example, Rae et al. (2014) examined the overlapping activity between action selection and action inhibition, with the latter referring to the externally-instructed go/no-go task, different from intentional inhibition paradigms considered here. Using clustering analysis, Zapparoli et al. (2017) reported regions separately associated with the *what, when* and *whether* components of intentional action. Our specific focus on free choice paradigms resulted in more studies from the literature search than previous meta-analyses: 35 experiments vs. 17 studies on intentional decision in Rae et al. (2014) and 15 studies in Zapparoli et al. (2017). With the increased power, the current study confirmed that the cortical activation pattern reported here is generalizable across a rich set of free-choice paradigms. It further confirmed the involvement of the AIC that was not consistently observed in previous studies.

The meta-analytic decoding analysis suggested that the spatial convergence of fMRI/PET responses in intentional decision is similar to previous experiments investigating certain cognitive terms, e.g., cognitive control, working memory and conflict. This is an exploratory analysis, and the decoding results were not tested against a null hypothesis to confirm the involvement of a particular cognitive function. Rather than interpreting cognitive functions of a single brain region, the meta-analytic decoding analysis quantitatively related whole-brain ALE results of intentional decision to previous imaging results. The overlapping brain maps between intentional decision and other cognitive paradigms, as suggested by our exploratory analyses, may lead to the development of new hypotheses and predictions. For example, one may test the interplay between intentional choices and working memory, with the expectation that lesion or brain stimulation to a region associated with both processes would affect behaviour. Therefore, our results provide insight into the cognitive roles of brain networks that mediate intentional behaviour in humans, which we discuss below together with their potential computational processes.

### 4.1 Functional localization of intentional decision in the brain

The brain areas involved in intentional decision overlap closely with the multiple demand network (Duncan and Owen, 2000; Duncan, 2010), a “task-positive” co-activation pattern associated with diverse cognitive demands (Fox et al., 2005; Dosenbach et al., 2006). A closer inspection of the literature indicates that subcomponents of this network may serve different cognitive roles during intentional decisions, which is also supported by our meta-analytic decoding results (Fig. 4).

A large body of evidence indicates the putative role of ACC in conflict monitoring (Botvinick et al., 2004). Conflicts in information processing arise from the presence of response competition. Greater ACC activation is consistently observed when (1) one or more prepotent responses need to be overridden, such as in the Stroop task (MacLeod and MacDonald, 2000; Barch, 2001) and the flanker task (Botvinick et al., 1999; Bunge et al., 2002), or (2) a voluntary choice is needed among multiple underdetermined options, like in all the free-choice paradigms discussed here. Although the existing literature of conflict monitoring is largely focused on the ACC, the adjacent pre-SMA is also sensitive to the presence of conflict, in particular the conflict in response selection, as lesions in this region lead to deficits in exerting voluntary control over immediate actions (Nachev et al., 2007). According to the conflict monitoring theory, as multiple options become available in the free-choice paradigm, increased ACC and pre-SMA activities may encode conflicts as an index of the need for greater cognitive demand, which in turn trigger voluntary choices to reduce or resolve the conflict (Botvinick et al., 2004; Botvinick, 2007). A direct prediction of this proposition is that the activity in the medial prefrontal cortex should increase proportionally, at least to some extent, to the number of available options in the free-choice paradigm, which has been validated in previous studies (e.g., Forstmann et al., 2006).

ACC, more specifically the dorsal ACC, is also involved in a diverse set of cognitive functions beyond conflict monitoring, including error detection and response selection (Bush et al., 2000; Paus, 2001). Using a modified flanker task with free choices, Lau et al. (2006) showed a disassociation between ACC and pre-SMA in conflict monitoring and response selection, respectively. However, there is a lack of studies to attribute a definitive functional role to the dorsal ACC across multiple free-choice paradigms.

Beyond the medial prefrontal cortex, the frontoparietal network on the lateral brain surface has a distinct functional connectivity pattern relating to cognitive control (Corbetta and Shulman, 2002) and executive task performance (Seeley et al., 2007). Two functions of this network are essential to intentional behaviour. First, intentional decisions in the free-choice paradigm are, by definition, rendered endogenously. Nevertheless, the brain may still establish a “task set” that incorporates transient and arbitrary rules in addition to relevant exogenous information, such as associations of stimuli and imagined outcomes as well as available options (Sakai, 2008). Both single-unit recording in non-human primates (Quintana and Fuster, 1999; Asaad et al., 2000; Wallis et al., 2001) and brain imaging in humans (Bunge et al., 2002; Sakai and Passingham, 2003) have identified neural representations of various task sets in the frontoparietal network. The encoding of a task set can be actively maintained in this network until its execution (Zhang et al., 2013), thereby facilitating the intentional decision process to unfold in time. Second, intentional behaviour is commonly accompanied by the subjective experience of volitional control (Haggard, 2008), which requires internal models that matches the consequences of the response against its initial intention (Wolpert et al., 1995). It has been proposed that the parietal cortex hosts such internal models (Desmurget and Grafton, 2000), as patients with parietal lesions exhibited altered behavioural and electrophysiological signatures of their intention to act (Sirigu et al., 2004).

Our meta-analysis across all free-choice experiments showed the consistent involvement of the AIC during intentional decision, in spite of the lack of significant insula activity in some studies (e.g., Van Eimeren et al., 2006). This supports an earlier account that the AIC is a key component of the integrated brain network involved in intentional behaviour (Brass and Haggard, 2010). Anatomically, the AIC connects directly to the ACC (Augustine, 1996; Moisset et al., 2010). Functionally, robust coactivation in the AIC and ACC was observed across multiple cognitive domains (Medford and Critchley, 2010) as well as in resting-state (Chang et al., 2013), and both regions are a part of the salience network (Chen et al., 2016). It may therefore be tempting to ascribe the AIC activity to conflict processing during intentional decision, similar to that of ACC. An alternative proposal originated from the AIC's unique function in signalling introspective awareness (Craig, 2009) or subjective salience (Menon and Uddin, 2010) of cognitive (Preuschoff et al., 2008), homeostatic (Craig et al., 2000; Farrer and Frith, 2002) and emotional (Jabbi et al., 2007) information, which is not shared with the ACC. According to this theory, AIC activity reflects the affective consequences of intentional decisions. In other words, the AIC may not directly associate with the formation of current intention; instead, it evaluates the outcome of the intentional act with respect to an internal model of one's long term goal (see Brass and Haggard, 2010 for a detailed review).

### 4.2 The which, when and whether components of intentional behaviour

The current study does not aim to support or challenge the conceptual Which-When-Weather (WWW) distinctions (Brass and Haggard, 2008). Instead, we focused on a common contrast in free-choice paradigms (i.e., choice vs. specified), which emphasizes the presence of an intentional choice among alternatives. Our literature review suggested that papers reporting this contrast examined the BOLD/PET response to decisions between actions (reactive intention), between perceptual targets (perceptual intention), between act and stop (inhibitory intention) and between more complex cognitive operations (cognitive intention). Main research questions in these paradigms naturally involve the *which* and *whether* components of the WWW model.

The timing of intentional behaviour, the *when* component of the WWW model, is initially and commonly investigated using electrophysiology (Libet, 1985), focusing on the time course of neural activity leading to an intentional act. This does not preclude the use of fMRI/PET to study the contrast of responses at intentional timing vs. responses at specified timing.

Imaging studies on the *when* component employed diverse designs and their primary contrasts includes: (1) subjective awareness of the timing of intention as in Libet's task (Libet et al., 1983) vs. subjective awareness of the timing of an action (Lau et al., 2004a); (2) free to act at any time vs. act after a response cue (Hoffstaedter et al., 2013); (3) free to act at one of several time points vs. act at a specified time point (Krieghoff et al., 2009; Zapparoli et al., 2018). As such, existing studies considering the *when* component cannot be readily formalized as a choice between one overt option versus another, different from other free-choice paradigms considered here. Also, previous meta-analysis has specifically summarized studies of the *when* component (Zapparoli et al., 2017). Hence, we did not include those papers in the current meta-analysis. We outlined below how the *when* component can be incorporated into a computational model of intentional decision.

### 4.3 Computational processes of intentional decision

With the identification of the consistent brain network for intentional decision-making, a new question arises: what is the computational process underlying intentional decision? Converging findings from behavioural modelling (Ratcliff, 2006), single-unit recoding (Kim and Shadlen, 1999; Shadlen and Newsome, 2001; Mazurek et al., 2003) and imaging (Heekeren et al., 2004; Ploran et al., 2007; Ho et al., 2009) experiments suggest that, when making choices based on external stimuli, an accumulation-to-threshold mechanism governs the decision-making process (Smith and Ratcliff, 2004; Gold and Shadlen, 2007; Heekeren et al., 2008): the evidence supporting one or multiple options are accumulated over time, and a choice is made when the accumulated evidence reached a decision threshold. For perceptual decisions with noisy sensory stimuli, this accumulation process reduces the momentary noise in information-processing and in turn results in more accurate decisions (Bogacz et al., 2006, 2007; Zhang and Bogacz, 2010).

For intentional decisions, it has been shown that a computational model implementing the accumulation-to-threshold mechanism can well describe the behavioural performance (i.e., response time distributions and choice probabilities) of both RI (Zhang et al., 2012) and PI paradigms (Zajkowski et al., 2020). Furthermore, the accumulated evidence predicted by the model is associated with the BOLD response in the ACC and pre-SMA on a trial-by-trial basis (Zhang et al., 2012). These results raise an intriguing possibility that, during intentional decision, the medial prefrontal cortex implements the accumulation-to-threshold process to integrate over time the transitory intention of choosing different options, until the accumulated intention for one choice reaches a decision threshold.

This hypothesis is supported by several electrophysiological studies, which characterised the accumulation process during intentional behaviour at a high temporal resolution. First, in Libet's paradigm of voluntary action, the readiness potential measured by scalp EEG precedes participants’ conscious awareness of their voluntary intention (i.e., the “urge to move”, Libet et al., 1983). An accumulator model can be fit to the time latency of participants’ urge to move, and the activity of the accumulator qualitatively reproduces the time course of the readiness potential prior to conscious intention (Schurger et al., 2012). Second, in a free-choice version of Libet's paradigm, when participants made intentional decisions between responding with their left or right hands, neural activity in the medial prefrontal cortex build up several hundred milliseconds before the onset of conscious intention (Fried et al., 2011). Further, medial prefrontal neurons contralateral to the acting hand exhibited larger activity than ipsilateral neurons (Fried et al., 2011). Therefore, the medial prefrontal cortex may host accumulated intentions of multiple responses as well as their mutual competition, from which voluntary acts are rendered via the accumulation-to-threshold mechanism.

The putative role of the medial prefrontal cortex in intention accumulation is not inconsistent with this region's function of conflict monitoring discussed above, because more free options would be associated with larger accumulated intention across alternatives as well as higher conflict. In this regard, intention accumulation can be interpreted as a computational implementation of detecting and resolving conflicts among underdetermined options. Therefore, we consider the accumulation process as a parsimonious computational framework for intentional behaviour outlined by the conceptual *what*-*when*-*whether* model (Brass and Haggard, 2008), because accumulator models can explain quantitatively both “*what”* (i.e., choice probabilities) and *“when”* (i.e., response time distributions) components. Interestingly, accumulator models can also be fitted to behavioural performance in externally-triggered stopping tasks (Gomez et al., 2007; Zhang et al., 2016). Future research should investigate if accumulator models can incorporate the “*whether*” component, or voluntary stopping in the II paradigm.

### 4.4 Paradigm-specific activations during intentional decision

By categorizing free-choice studies into different types according to their experimental design, we identified brain regions associated with consistent and specific convergence between studies on sub-categories of intentional decision (Fig. 3). The conjunction meta-analysis of the RI and PI paradigms showed that the pre-SMA/ACC and DLPFC are associated with both types of intentional decision. This is expected, as the RI and PI paradigms have a similar task structure, involving rapid voluntary choices among multiple action plans (Table 4). However, the contrast analysis did not reveal any difference between the two paradigms.

The II paradigm includes a unique option of not to act or intentionally inhibit one's action (Fig. 1C). The conjunction meta-analysis of the II and RI paradigms showed that the right supramarginal gyrus in the IPL and right pre-SMA was associated with both types of intentional decisions, and the contrast meta-analysis showed that the bilateral supramarginal gyrus was more likely to be activated in the II than that in the RI paradigm. In both RI and II paradigms, participants need to reprogram their response model according to available options in each trial, which fits the critical role of the supramarginal gyrus in action reprogramming (Hartwigsen et al., 2012). The same region is also sensitive to the content of action plans and their similarity (Quandt et al., 2017). It could be argued that options in the II paradigm are more dissimilar (i.e., acting versus stopping) than that in the RI paradigm (i.e., multiple but similar actions), which leads to the additional recruitment of the supramarginal gyrus in the II paradigm.

It is worth noting that the results of conjunction and contrast meta-analyses should be interpreted with caution, because of the limited number of studies available in each category. Our discussion on the functional roles involved in individual paradigms was based on previous evidence and hence was circumstantial. Furthermore, one potential confound of the contrast meta-analysis is that different paradigm categories may vary in their task difficulty, and hence the contrast between categories may not directly support the involvement of distinct cognitive processes. This issue can be examined in future studies that explicitly manipulate both task difficulty and intentional decision paradigms.

### 4.5 Future directions and conclusion

This analysis leaves open some issues for future research on human intentional decision-making. First, our systematic review identified only five studies in the CI category: three studies included options with attention shifts or saccades (Taylor et al., 2008; Jarvstad and Gilchrist, 2019; Ort et al., 2019), one with verbal responses (Frith et al., 1991) and the other one with arithmetic rules (Wisniewski et al., 2016). The small number of CI studies did not yield any significant result in the paradigm-specific meta-analysis, but that may reflect type II error. We recommend future research to explore different types of CI studies and examine the robustness and consistency of existing results across a range of distinct cognitive processes.

Second, our meta-analysis of the II paradigm did not show conventional regions involved in inhibitory control (Swick et al., 2011). We propose that this is due to the fact that our analysis used the contrast of intentional choice vs. specified response, with the former including intentional stopping and the latter including externally triggered stopping - this contrast may therefore not detect differential response inhibition. Indeed, the BOLD response in the AIC was higher during intentional stopping than intentional action execution (Brass and Haggard, 2007), while the inferior frontal gyrus is consistently observed during instructed stopping (Aron et al., 2004). To examine how the brain switches effectively between intentional and instructed stopping in the II paradigm, one need to examine the effective connectivity between these two regions and the medial prefrontal cortex, which is involved in both types of stopping (Kühn and Brass, 2009; Sharp et al., 2010).

Third, the current imaging literature on intentional behaviour indicates that the main focus is to localize associated brain regions or their underlying computational processes. Less is known about why a participant would intentionally choose one option over others in a trial. The answer to this question is important because the sequence of intentional decisions over trials are not completely random (Zhang and Rowe, 2015) but dependent on executive control of working memory (Baddeley et al., 1998), the context of a given choice in a sequence (Rowe et al., 2010), and other sources of response biases (Zajkowski et al., 2020). We suggest that the free-choice paradigm provides an ideal testbed for future research to investigate the interplay between the intentional process during a single trial and modulatory effects that operate at a longer time span.

Fourth, brain regions unlikely act in isolation during intentional decision, and the functional connectivity between regions cannot be ignored. Among all studies included in the current meta-analysis, only two examined functional connectivity (Rowe et al., 2005; Thimm et al., 2012), insufficient to draw inferences on a meta-analytic level. Using structural equation modelling, Rowe et al. (2005) showed that the prefrontal cortex has greater coupling with the motor cortex during free choices of actions, and with the visual cortex during free choices of colours. Similarly, using psychophysiological interaction analyses, Thimm et al. (2012) reported greater connectivity between the parietal cortex and the visual cortex during free choice of coloured targets. These results imply task-dependent changes in the effective connectivity between brain regions during free choices.

In conclusion, our meta-analysis identifies a brain network consistently activated when humans have the freedom to make intentional choices among multiple options. Some components of this network are recruited specifically in subcategories of the free-choice paradigm. Multiple cognitive and computational processes are involved in intentional decision, which collectively serve essential roles in shaping and maintaining volitional control.

## CRediT authorship contribution statement

**Ruoguang Si:** Conceptualization, Data curation, Formal analysis, Funding acquisition, Investigation, Methodology, Project administration, Visualization, Writing – review & editing. **James B Rowe:** Funding acquisition, Investigation, Writing – review & editing. **Jiaxiang Zhang:** Conceptualization, Formal analysis, Funding acquisition, Investigation, Methodology, Project administration, Supervision, Visualization, Writing – review & editing.

## Declaration of Competing Interest

The authors declare no competing financial interests.

## Appendix Supplementary materials

Image, application 1
