# Supplementary material for: Functional localization and categorization of intentional decisions in humans: A meta-analysis of brain imaging studies
Source: Neuroimage. 2021 Nov 15;242:118468. doi: 10.1016/j.neuroimage.2021.118468 (PMC8463837; doi:10.1016/j.neuroimage.2021.118468)
Supplement: Supplementary file 1 [file mmc1.docx]

**Functional localization and categorization of intentional decision in humans:
a meta-analysis of brain imaging studies**

**Supplementary Material**

Ruoguang Si, James B Rowe, Jiaxiang Zhang

**Supplementary Table 1.** Top 8 cognitive topics and their corresponding correlation coefficients with brain maps from the main (Figure 2) and paradigm-specific (Table 3) meta-analyses. The ranking is based on the meta-analytic decoding result of the main analysis of all studies (Figure 2).

| **Topics** | **ALL** | **RI** | **PI** | **II** |
| --- | --- | --- | --- | --- |
| Working memory | 0.4452 | 0.3934 | 0.4499 | 0.3072 |
| Task rules | 0.3920 | 0.3483 | 0.3250 | 0.2601 |
| Cognitive control | 0.3664 | 0.3108 | 0.3217 | 0.2711 |
| Conflicts | 0.3646 | 0.3502 | 0.3036 | 0.2520 |
| Attention | 0.3020 | 0.2798 | 0.2553 | 0.1383 |
| Imagery | 0.2710 | 0.2786 | 0.2335 | 0.1563 |
| Inhibition | 0.2658 | 0.2427 | 0.2086 | 0.2084 |
| feedback | 0.2505 | 0.2262 | 0.1572 | 0.2266 |

**Supplementary Table 2.** Full list of terms associated with the 8 topics in Supplementary Table 1. The number of studies of each topic was extracted from the Neurosynth database (Yarkoni et al., 2011, <https://neurosynth.org/analyses/topics/v4-topics-100/>).

| **Topics** | **Terms included** | **Num. Studies** |
| --- | --- | --- |
| Working memory | memory, working, task, load, verbal, maintenance, performance, cognitive, activation, information, tasks, term, capacity, probe, manipulation, executive, spatial, phase, encoding, updating, performed, storage, network, span, rehearsal, retention, increased, delay, function, accuracy, functions, demands, vwm, delayed, phonological, loads, demand, performers, sternberg, binding | 798 |
| Task rules | switching, set, rule, task, switch, rules, flexibility, shifting, sets, sorting, trials, shift, shifts, anxiety, switches, card, wcst, anxious, costs, paradigm, single, ef, required, wisconsin, stimulus, trial, worry, repeat, switched, paradigms, execution, depending, component, chunk, cost, gad, lower, updating, types, iu | 230 |
| Conflicts | conflict, interference, control, stroop, incongruent, task, response, congruent, olfactory, resolution, trials, odor, behavioral, attentional, color, cognitive, odors, simon, congruency, word, flanker, effect, monitoring, irrelevant, processing, activated, conflicting, incompatible, neutral, relevant, mechanisms, detection, conflicts, situations, resolve, adjustments, pre, compatibility, compatible, counting | 392 |
| Cognitive control | cognitive, control, performance, task, executive, function, functions, cognition, ability, attention, behavioral, tasks, functioning, test, goal, effort, individuals, behavior, demands, recruitment, abilities, lateral, performed, attentional, neuropsychological, directed, domains, tests, level, individual, relevant, behavioural, evidence, speed, performing, impairment, stroop, deficits, impaired, domain | 1098 |
| Attention | attention, attentional, visual, spatial, search, orienting, target, top, selective, control, location, areas, attended, network, stimulus, irrelevant, cues, distraction, shifts, relevant, feature, modulation, task, cued, mechanisms, color, cueing, focus, bottom, processing, attend, event, endogenous, cue, attending, allocation, directed, resources, modulated, perceptual | 841 |
| Imagery | wm, imagery, mental, imagined, rotation, mi, tasks, visual, visuospatial, motor, areas, spatial, ltm, imagination, imagine, transformation, image, imagining, mentally, images, activated, ability, angle, degrees, physical, strategy, manipulation, visuo, poor, actual, rotations, simulation, kinesthetic, gifted, representational, clock, angles, rehearsal, future, instructed | 355 |
| Inhibition | inhibition, response, control, inhibitory, stop, task, motor, signal, activation, trials, nogo, suppression, responses, successful, inhibit, behavioral, error, inhibited, inhibiting, pre, performance, prepotent, reactive, stopping, ability, sst, monitoring, suppress, correlates, gating, action, rifg, success, proactive, errors, tasks, required, participants, voluntary, behavior | 421 |
| Feedback | feedback, error, errors, learning, prediction, monitoring, correct, negative, performance, response, outcome, reinforcement, positive, trials, processing, outcomes, task, trial, reversal, behavior, stimulus, expected, signal, detection, signals, events, expectations, incorrect, expectation, predictive, contingencies, correlated, event, reward, contingency, adaptation, probability, adjustment, actual, unexpected |  |

**Supplementary Table 3.** The summary of behavioural performance reported in individual studies. Response accuracy was defined as the proportion of trials with (1) valid response from available options and (2) response time within the maximum time limit.

| **No.** | **Study** | **Reaction Time (ms)** | | | | **Accuracy (%)** | | | |
| --- | --- | --- | --- | --- | --- | --- | --- | --- | --- |
|  |  | **Specified** | | **Intentional** | | **Specified** | | **Intentional** | |
|  |  | Mean | SD | Mean | SD | Mean | SD | Mean | SD |
| 1 | (Beudel and De Jong, 2009) | 1500* | - | 1300* | - | - | - | - | - |
| 2 | (Deiber et al., 1991) | - | - | - | - | - | - | - | - |
| 3 | (Deiber et al., 1996) | 300* | - | 300* | - | 97^#^ | - | 99^#^ | - |
| 4 | (François-Brosseau et al., 2009) | 739 | 40 | 504 | 60 | 99.1^#^ | 0.4 | 98.2^#^ | 1.3 |
| 5 | (Frith et al., 1991) | - | - | - | - | - | - | - | - |
| 6 | (Gerardin et al., 2004) | 617 | 80 | 571 | 63 | 98.89^#^ | 0.7 | - | - |
| 7 | (Hoffstaedter et al., 2013) | 412 | 124 | 436 | 77 | 98.49^#^ | 1.87 | - | - |
| 8 | (Hyder et al., 1997) | - | - | - | - | - | - | - | - |
| 9 | (Krieghoff et al., 2009) | - | - | - | - | 99.15 | 1.11 | - | - |
| 10 | (Mueller et al., 2007) | 569 | 39.59 | 501 | 70.60 | - | - | - | - |
| 11 | (Rae et al., 2014) | 607 | - | 634 | - | 94^#^ | - | - | - |
| 12 | (Rowe et al., 2010) | - | - | - | - | - | - | - | - |
| 13 | (Schouppe et al., 2014) | - | - | - | - | - | - | - | - |
| 14 | (Van Eimeren et al., 2006) | - | - | - | - | 98 | - | - | - |
| 15 | (Bode et al., 2013) | - | - | - | - | 88.35^ | - | - | - |
| 16 | (Filevich et al., 2013) | 1950* | - | 1900* | - | - | - | - | - |
| 17 | (Forstmann et al., 2006) | 829 | - | 874 | - | - | - | - | - |
| 18 | (Lau et al., 2004b) | 952.9 | 117.1 | 1280.1 | 335.1 | - | - | - | - |
| 19 | (Orr and Banich, 2014) | - | - | - | - | - | - | - | - |
| 20 | (Rens et al., 2018) | - | - | - | - | 98.5 | 3.6 | - | - |
| 21 | (Rowe et al., 2005) | - | - | - | - | - | - | - | - |
| 22 | (Rowe et al., 2008) | 935.4 | 24.65 | 920.6 | 24.5 | - | - | - | - |
| 23 | (Thimm et al., 2012) | 666 | 66 | 818 | 110 | 97^#^ | 3 | 96.7^#^ | 3.9 |
| 24 | (Dall’Acqua et al., 2018) | 439.7^ | 88.3^ | - | - | - | - | - | - |
| 25 | (Karch et al., 2010b) | 424.2 | 87.49 | 647.9 | 161.53 | 98.1 | 1.16 | - | - |
| 26 | (Karch et al., 2010a) | 404.8 | 79.9 | 575.4 | 105.4 | 96.5 | 3.86 | - | - |
| 27 | (Karch et al., 2009) | 473.9 | 142.23 | 689.5 | 187.54 | 98 | 1.25 | - | - |
| 28 | (Lynn et al., 2016) | 597 | - | 903 | - | - | - | - | - |
| 29 | (Omata et al., 2019) | 9615^ | 360^ | 8880^ | 1545^ | - | - | - | - |
| 30 | (Schel et al., 2014) | 301 | 39 | 372 | 89 | 96.46 | - | - | - |
| 31 | (Frith et al., 1991) | - | - | - | - | - | - | - | - |
| 32 | (Jarvstad and Gilchrist, 2019) | 251 | 50 | 257 | 80 | - | - | - | - |
| 33 | (Ort et al., 2019) | - | - | - | - | 93.88^ | - | - | - |
| 34 | (Taylor et al., 2008) | 490* | - | 480* | - | 91 | - | 85 | - |
| 35 | (Wisniewski et al., 2016) | - | - | - | - | - | - | 96.6^#^ | 0.4 |

* The reaction time was not reported in the text but estimated from figures.

^ The experiment reported the reaction times or accuracy of different choices separately (e.g., go and no-go behaviours). The results in this table were the averaged reaction time or accuracy across all choices.

^#^ The accuracy was not directly reported but calculated from the error rate. Accuracy = 100% - error rate.
